# Correlates of smoking susceptibility among adolescents in a peri-urban area of Nepal: a population-based cross-sectional study in the Jhaukhel-Duwakot Health Demographic Surveillance Site

**DOI:** 10.3402/gha.v7.24488

**Published:** 2014-07-16

**Authors:** Umesh R. Aryal, Max Petzold, Göran Bondjers, Alexandra Krettek

**Affiliations:** 1 Department of Community Medicine, Kathmandu Medical College, Kathmandu, Nepal; 2 Department of Internal Medicine and Clinical Nutrition, Institute of Medicine, Sahlgrenska Academy, University of Gothenburg, Gothenburg, Sweden; 3 Akademistatistik – Centre for Applied Biostatistics, Occupational and Environmental Medicine, Sahlgrenska Academy, University of Gothenburg, Gothenburg, Sweden; 4 Sahlgrenska Academy, University of Gothenburg, Gothenburg, Sweden; 5 Nordic School of Public Health NHV, Gothenburg, Sweden

**Keywords:** adolescents, peri-urban, susceptibility to smoking, sociodemographic factors, environmental factors

## Abstract

**Background:**

Susceptibility to smoking is defined as an absence of firm commitment not to smoke in the future or when offered a cigarette by best friends. Susceptibility begins in adolescence and is the first step in the transition to becoming an established smoker. Many scholars have hypothesized and studied whether psychosocial risk factors play a crucial role in preventing adolescent susceptibility to smoking or discourage susceptible adolescents from becoming established smokers. Our study examined sociodemographic and family and childhood environmental factors associated with smoking susceptibility among adolescents in a peri-urban area of Nepal.

**Design:**

We conducted a population-based cross-sectional study during October–November 2011 in the Jhaukhel-Duwakot Health Demographic Surveillance Site (JD-HDSS) located in a peri-urban area near Kathmandu, the capital city of Nepal, where tobacco products are easily available. Trained local enumerators conducted face-to-face interviews with 352 respondents aged 14–16. We used stepwise logistic regression to assess sociodemographic and family and childhood environmental factors associated with smoking susceptibility.

**Results:**

The percentage of smoking susceptibility among respondents was 49.70% (95% CI: 44.49; 54.93). Multivariable analysis demonstrated that smoking susceptibility was associated with smoking by exposure of adolescents to pro-tobacco advertisements (AOR [adjusted odds ratio] =2.49; 95% CI: 1.46–4.24), the teacher (2.45; 1.28–4.68), adolescents attending concerts/picnics (2.14; 1.13–4.04), and smoking by other family members/relatives (1.76; 1.05–2.95).

**Conclusions:**

Smoking susceptible adolescents are prevalent in the JD-HDSS, a peri-urban community of Nepal. Several family and childhood environmental factors increased susceptibility to smoking among Nepalese non-smoking adolescents. Therefore, intervention efforts need to be focused on family and childhood environmental factors with emphasis on impact of role models smoking, refusal skills in social gatherings, and discussing harmful effects of smoking with family members and during gatherings with friends.

The World Health Organization (WHO) Framework Convention on Tobacco Control (WHO-FCTC) and MPOWER Policies aim to protect people's health through key interventions (1, 2). These intervention programs seek to combat tobacco-related morbidity, mortality, and economic losses by restraining smoking initiation among children, adolescents, and young adults and also by promoting smoking cessation among adults (2). The Government of Nepal has signed the FCTC and established tobacco control polices and laws that combat the use of tobacco products. However, these policies remain ineffective due to limited resources and poor implementation (3, 4).

Smoking initiation among adolescents is progressing through a sequence of phases, including preparation, contemplation, trier, experimenter, and regular and established smoker (5). In the preparation stage, non-smoking adolescents are susceptible to smoking if they have opportunity to smoke and lack a strong commitment not to smoke in the future or if offered a cigarette by friends (6). Susceptibility to smoking is a cognitive shift during the preparation stage that precedes experimentation with cigarettes (6, 7). Most young children are committed not to try smoking (7). However, during adolescence they begin to think that they may try to smoke sometime in the future. When adolescents have the opportunity to try smoking, they will not refuse to smoke as they reassess the information about acceptability of cigarettes and the expected consequences of smoking (7). This leads adolescents to be more vulnerable to pro-tobacco influences. Thus, a cognitive shift occurs from resistance to ambivalence which defines susceptibility and strongly predicts experimentation with smoking (6, 7). Therefore, susceptibility to smoking is an important construct in smoking research as it is an early stage of cognitive change among adolescents that ultimately results in experimentation with smoking and to adolescents becoming established smokers (6, 7).

Some studies have used well-known theories of health behavior to explain the psychosocial risks and protective factors that influence adolescents’ decisions to initiate or refuse smoking (8–11). The link between smoking susceptibility and psychosocial risk factors (e.g. socioeconomic, environmental, behavioral, and personal factors) among adolescents was examined predominantly in the United States (12–14) and little evidence is available to date from low and middle-income countries (15–17).

Due to increased smoking and alcohol consumption, Nepal faces a growing burden of non-communicable diseases (NCDs) (18). Indeed, NCDs now account for 50% of all deaths annually, an increase of 8% compared to a decade ago (18, 19). Most premature deaths among adults are attributed to risky behavior patterns like smoking that emerge during mid-adolescence (14–15 years) (20, 21). The use of tobacco provides an opportunity to participate in a behavior that defies established social norms (22). Nepalese cross-sectional studies among adolescents and youths show that the average age of smoking initiation is 13–16 years (23–26). According to the Global Youth and Tobacco Survey (GYTS), 10% of in-school adolescents have smoked at least once and 16% of non-smoking adolescents (13–15 years) would like to initiate smoking within a year (27). Our recent community-based study in the Jhaukhel-Duwakot Health Demographic Surveillance Site (JD-HDSS) demonstrates that non-smoking adolescents who perceive social benefits and no addiction risk of smoking are more likely to initiate smoking compared to those who perceive short-term risks (28). The current study highlights how susceptible adolescents differ from their non-susceptible counterparts. To our knowledge, earlier Nepalese studies are limited to the exploration of social and demographic factors associated with tobacco use among in-school adolescents and a comparison of tobacco users versus nonusers (24, 25, 29). However, intervention programs in Nepal will benefit from a better understanding of risk factors that associate with smoking susceptibility among adolescents. Therefore, the present study examined the sociodemographic and family and childhood environmental factors associated with smoking susceptibility in 14–16-year-old Nepalese adolescents.

## Methods

### Study design and setting

We conducted a population-based cross-sectional study during October–November 2011 in the JD-HDSS, a peri-urban area located in the Bhaktapur district near Kathmandu, the capital city of Nepal (30). JD-HDSS was established in 2010 as collaboration between the University of Gothenburg and the Nordic School of Public Health NHV, Sweden as well as Kathmandu Medical College and Nepal Medical College, Nepal, to monitor demographic and health data in close proximity to the community hospitals run by the collaborative partners (30). The site is in an urbanizing area that is rapidly moving towards an urban lifestyle with all modern facilities. Adolescents have easy access to tobacco products and are exposed to advertising. According to the 2011 national census, there were 304,651 inhabitants in the Bhaktapur district of which 5% people lived in the Jhaukhel and Duwakot villages (31). We conducted a baseline survey in 2010 during the establishment of JD-HDSS and found that the study area encompassed 2,712 households (1,155 in Jhaukhel and 1,557 in Duwakot) and 13,669 individuals (6,057 in Jhaukhel and 7,612 in Duwakot). There were 2,776 adolescents aged 10–19 of which 909 were adolescents aged 14–16 who are vulnerable to smoking (30).

### Sampling techniques and sample size

**Fig. 1:**
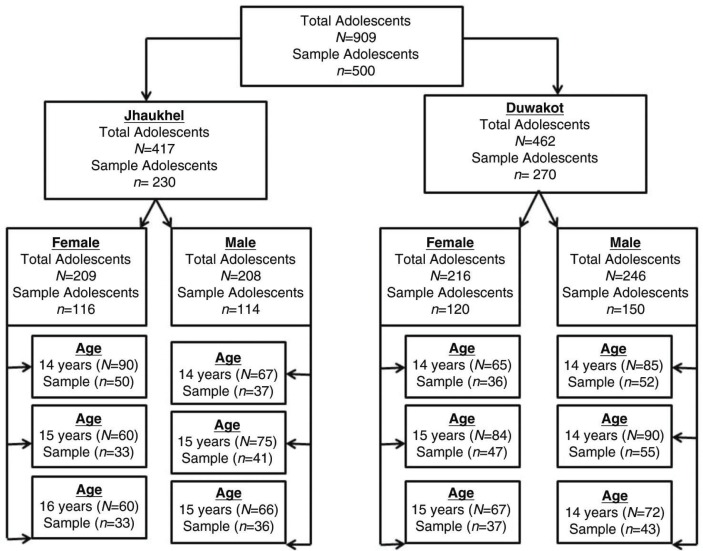
Process of sampling techniques. In Step 1, we obtained a sampling fraction that represented 49.8 and 55.5% of male adolescents from Jhaukhel and Duwakot (i.e. 114 males from Jhaukhel and 150 males from Duwakot). In Step 2, we further classified the sex of the adolescents into three age groups (14-, 15-, and 16-year-olds) for each village. Among the 114 male respondents in Jhaukhel, 32.2, 36.1, and 31.7% belonged to the 14-, 15-, and 16-year-old age groups, respectively. Among 116 female respondents, 42.9, 28.6 and 28.6% belonged to the same age groups, respectively. Among 150 male respondents in Duwakot, 34.5, 36.4, and 29.1% belonged to the 14-, 15-, and 16-year-old age groups, respectively; 30.4, 38.7 and 30.9% of the 120 female respondents belonged to the same age groups, respectively. Finally, we used systematic sampling from each age group to select the adolescents. During analysis, all missing cases and ‘I do not know’ answers were excluded and analysis was done for 352 respondents.

The sampling techniques and sample size calculation have been described in detail earlier (28). We used proportionate stratified sampling to select 500 respondents, taken from 909 adolescents aged 14–16 living in the JD-HDSS (30). Figure 1 explains how 500 respondents were selected for the current study. Stratified sampling improved the representation of particular minority groups within the population (having less number of units in population sex and age wise), prevented oversampling of the respondents as well as made it possible to make valid inferences from the sample to the population (32). The minimum sample size of 385 respondents was calculated based on unknown prevalence of smoking (50% assumed for conservative sample size estimates), absolute precision 5%, incomplete questionnaires, and 95% confidence limits (33). Assuming 20% non-response rate and incomplete questionnaires, we decided to include 500 adolescents. Five hundred adolescents were included according to the following steps: 1) the total adolescent population was divided into two groups according to area (Duwakot and Jhaukhel); 2) area-wise adolescents were grouped by sex; 3) each sex (male or female) was further classified into three age groups (14-, 15-, and 16-year-olds). Then to select the adolescents from each age group, systematic sampling was used as it was assumed that the population units do not follow any pattern (say only smokers) that the sampling interval was different for each group (32). For example, in our study the sampling interval was nearly 2. Using a sampling fraction, the required number of adolescents was selected in each step.

### Data collection

We collected data via face-to-face interviews using a semi-structured questionnaire based on the GYTS 2007 (34), the Teen Smoking Questionnaire (35), perceived risk and benefit items from Halpern-Felsher et al. and Song et al. (36, 37), and a report by the US surgeon general (38). Our questionnaire was adapted to the cultural context of Nepal and pretested in Chagunarayan, a village that exhibits population characteristics similar to JD-HDSS.

Trained local enumerators visited the respondents’ households and conducted 60-min interviews at a time convenient for each respondent. Collected information included sociodemographic characteristics, smoking activities of family members/relatives/teachers/friends, exposure to media and advertising related to tobacco and NCD education (i.e. anti-smoking messages and school curriculum), perception of smoking-related risks and benefits, adolescents’ smoking behavior, smoking cessation, and health status. Enumerators were supervised by field supervisors, a field coordinator, and a PhD student (URA). The field supervisors were responsible for spot-checking and discussing field site issues with the field coordinator and PhD student to ensure maximum response rates. To detect errors and ensure the completeness of our data, we randomly checked completed questionnaires both in the field and at the JD-HDSS office before data entry. Erroneous forms were returned to the field for renewed data collection. Prior to data entry, data entry operators were given 3 days training under the supervision of the PhD student.

### Study variables

#### Dependent variable

Enumerators asked three questions related to the transition from non-smoking to smoking susceptibility (6):

- Will you try a cigarette (taking even just one puff) sometime in the next 6 months? Response options were 1) definitely will not, 2) may not, 3) maybe will, and 4) definitely will
- If one of your best friends offers you a cigarette, do you smoke? Response options were 1) never, 2) sometimes, and 3) always
- Do you think you will smoke cigarettes 5 years from now? Response options were 1) not at all, 2) slightly likely, 3) moderately likely, 4) very likely, and 5) most likely.

At first, all responses were treated as continuous data (1, 2, 3, 4 and 5) and found to be skewed (Mean>Median, the value of skewness is greater than 2) (39, 40). Since the data were skewed, we classified each question into two groups and treated them as binary variable, that is, not susceptible to smoking and susceptible to smoking based on the median value (1). Respondents who answered ‘definitely will not/never/not at all’ were considered not susceptible to smoking and coded as 0. All other response options were considered susceptible to smoking and coded as 1. The binary variable enables us to compare proportion differences in adolescents’ characteristics that are exposed to different risk factors of smoking initiation. Cronbach alpha, which measures the internal consistency, was 0.6. Prior to defining smoking susceptibility, never smoking was defined by those answering ‘no’ (response option: yes/no) to ‘Have you ever (even a few puffs) smoked cigarettes?’ The measure was similar to that reported by Pierce et al., but we adopted the questions to the local context so that adolescent felt comfortable responding (6). Further, adolescents who answered questions related to smoking susceptibility were denoted responders, otherwise they were non-responders.

#### Independent variables

##### Sociodemographic variables

Table 1 shows the independent sociodemographic variables included in this study.

**Table 1:** List of sociodemographic variables included in the study

Categories	Sub-categories
Age	14–16 years
Sex	Male/female
Ethnicity (41)	Upper caste groups (Brahmin, Chhetri, and Thakuri)
	Relatively advantaged group (Newar)
	Indigenous disadvantaged groups (Magar and Tamang)
	Socioeconomically disadvantaged group (Dalits)
Education status (43)	Primary level (grades 1–5)
	Lower secondary level (grades 6–8)
	Secondary level (grades 9–10)
Wealth Index (44)	Lowest, second, middle, fourth, upper
Father's occupation (42)	Service, business, farmer, retired, or unemployed
Mother's occupation (42)	Service, agriculture, housework, or business
Literacy status of parents (43)	Literate/illiterate
Type of family	Nuclear (father/mother/children living together)
	/Joint (father/mother/children/uncle/anti/grandfather/mother, etc. living together)
Monthly out-of-pocket expenditures (Nepalese rupees (NPR))	Based on median

We adopted the definition of categorical variables from reports by the Government of Nepal and previous publications and reports (33, 41–45).

##### Wealth index

This index, a proxy measure for the respondents’ economic status based on information on household ownership of assets (radio, bicycle, television, refrigerator, motorbike, washing machine, computer, and car), was constructed using principal component analysis (44). It is an indicator of the level of wealth (i.e. lowest quintile to highest quintile) that is consistent with measures of expenditures and income. The index has been widely used and tested in many countries in relation to inequalities in household income.

##### Literacy definition

We defined literate as a person aged 15 and above who could read, write, and do simple computation (43).

##### Family and childhood environmental variables

We assessed parental smoking; sibling smoking; smoking habits of other family members (uncle, aunt, grandfather, grandmother, etc.); asked to bring cigarettes from shop; asked to light cigarettes; exposure to secondhand smoke; involvement in extracurricular activities (e.g. quiz, debate); attending concerts/picnics with friends; exposure to pro-tobacco advertisements; exposure to anti-smoking messages; and whether respondents had discussed harmful effects of tobacco smoking with family members. We adapted and modified the definition of these variables to the Nepalese context (34, 38).

Response categories included 1) a lot/a few/none, 2) yes/no/do not know or not sure, 3) sometimes/most times/never, and 4) sometimes/always/never. For analysis, the response options ‘no/never/few’ were coded as 0 and the remaining items were coded as 1. We excluded the response options ‘do not know’, ‘not sure’, and missing answers from the analysis.

### Statistical analysis

Data was entered in EpiData version 3.1 and analyzed with SPSS version 17.We computed median and interquartile ranges for skewed numerical data and percentages for categorical variables. To measure the association between smoking susceptibility and explanatory variables (i.e. sociodemographic and environmental factors), we performed logistic regression analysis at both the univariate and multivariable levels. We entered all factors that revealed a significant difference in the univariate analysis into stepwise multivariable regression analysis. We performed stepwise multivariable logistic regression to identify the most parsimonious set of independent variables that are effective in explaining the dependent variable. Data are presented as unadjusted odds ratios (OR) and adjusted odds ratios (AOR) with 95% confidence interval (CI) from univariate and multivariable logistic regression analyses, respectively. Both Chi-square and Fisher exact test were used to identify the proportion differences between two groups. When expected frequencies were less than 5 in univariate analysis, we computed the exact 95% CI. Since most independent variables are binary in our study, we performed the following steps to check collinearity: 1) we looked for OR for each independent variable by pretending one of them is an outcome (exposure to second hand smoking); 2) we checked what happens to the regression coefficients and standard error when the number of variables are entered in the logistic regression model; 3) finally, the models were evaluated through maximizing adjusted *R* ^2^ (45). The level of significance was set at alpha (*α*)=0.05.

We also computed a cumulative risk score for each respondent by summing the significant risk factors identified from the multivariable logistic regression. All risk factors were dichotomous in nature (i.e. whether the respondents were exposed to a particular factor or not) (46). We then assigned each risk factor the value of 1 and created a risk index by summing risks. Cumulative risk scores ranged between 0 and 4. Only 14 respondents scored 0, among which 10 were not susceptible to smoking. For analysis purposes, we combined scores 0 and 1 and classified the combination as ≤1. We kept the remaining scores separately as 2, 3, and 4. Finally, we computed ORs using univariate logistic regression.

### Ethical considerations

The Nepal Health Research Council and the Ethical Committee of Kathmandu Medical College granted ethical approval of this study. We also acquired permission from local leaders and authorities. We separately obtained informed assent from respondents under 18 years of age and informed consent from their parents. We explained the objectives of the study to parents, informing them that participation was voluntary, responses would not be disclosed, and respondents’ privacy would be maintained. After each interview, we gave respondents an information leaflet about the harmful effects of smoking.

## Results

Among 500 randomly selected adolescents, two were excluded from the study (one refused to participate and one had impaired hearing), 13 were smokers, and 485 were non-smokers. All 13 smokers were male and most of them were aged 16. We excluded 133 non-smokers because they did not respond to questions related to smoking susceptibility. The final sample included 352 respondents who had never smoked a cigarette, not even a puff, prior to the survey. Further, we classified 485 non-smokers into two groups, that is, responders (352 non-smoking adolescents) and non-responders (133 non-smoking adolescents) to compare their characteristics. Non-responses were higher among females and had less monthly out-of-pocket expenditure [i.e. ≤NPR 600 ($7.50)] (Appendix 1). The non-responders reported that they had few smoker friends. They were less exposed to anti-smoking messages at school and participated less in concerts/picnics (Appendix 2).

The inclusion criteria for this study were all respondents living in the JD-HDSS, who were enlisted within the sampling frame, who were aged 14–16 during the survey period and who were willing to participate voluntarily. Otherwise, adolescents were excluded from analysis.

### Sociodemographic characteristics

General demographic characteristics of the respondents have been described earlier (38). Table 2 shows additional characteristics. The fathers of nearly 42% of respondents were service holders, 29.3% were farmers, and 26.8% were working in business; remaining fathers were either retired or unemployed. A majority (63.8%) of the respondents’ mothers performed housework. Two out of three respondents were studying at secondary level. Based on household asset scores, 65% of respondents belonged to the lower class, 33.5% to the middle class and 1.5% to the upper class. The monthly median for out-of-pocket expenditure by respondents was NPR 600 ($7.50) and the interquartile range was NPR 400–900 ($5.00–$11.25).

**Table 2:** Sociodemographic factors and potential association with smoking susceptibility among adolescents

		Susceptibility to smoking (*n*=175)	Non-susceptibility to smoking (*n*=177)		Unadjusted odds ratio (OR)
				
Variables	Responses	Number (%)	Number (%)	*P*^a^	95% (CI)
Sex	Female	70 (43.5)	91 (55.5)		1 (Ref)
	Male	105 (55)	86 (45)	0.03	**1.58 (1.04–2.42)**^b^
Age	16	47 (43.9)	60 (56.1)		1 (Ref)
	15	63 (52.5)	57 (47.5)	0.35	1.41 (0.91–1.41)
	14	65 (52.0)	60 (48.0)		1.38 (0.8–2.40)
Ethnic group	Upper caste	89 (45.4)	107 (54.6)		1 (Ref)
	Relatively advantaged	74 (55.6)	59 (44.4)	0.29	1.51 (0.95–2.41)
	Indigenous and socially disadvantaged	12 (52.2)	11 (47.8)		1.31 (0.50–3.38)
Education status (Grade)	Higher secondary (11–12)	42 (46.7)	48 (53.3)		1 (Ref)
	Secondary (9–10)	115 (50.2)	114 (49.8)	0.77	1.15 (0.53–3.24)
	Lower secondary (6–8)	16 (53.3)	14 (46.7)		1.31 (0.50–3.38)
Wealth index (Quintile)	Lowest	40 (51.9)	37 (48.1)		1 (Ref)
	Second	37 (56.9)	28 (43.07)		0.95 (0.52–1.82)
	Middle	24 (58.5)	41 (41.5)	0.06	0.78 (0.39–1.50)
	Fourth	38 (50.6)	37 (49.4)		1.76 (0.88–3.50)
	Upper	35 (50.7)	34 (49.3)		1.00 (0.52–1.92)
Father's occupation	Service	70 (48.9)	73 (51.1)		1 (Ref)
	Business	42 (51.2)	40 (48.8)		1.02 (0.59–1.76)
	Farmer	50 (49.5)	51 (50.5)		1.09 (0.61–1.96)
	Retired/unemployed	12 (63.2)	7 (36.8)	0.25	1.79 (0.67–5.37)
Mother's occupation	Service	6 (35.3)	11 (64.7)		1 (Ref)
	Agriculture	36 (46.6)	41 (53.4)	0.44	1.02 (0.59–1.76)
	Housework	114 (50.9)	110 (49.1)		1.09 (0.61–1.96)
	Business	19 (57.6)	14 (42.4)		1.79 (0.67–5.37)
Father's literacy status^c^	Literate	173 (50.9)	167 (49.1)		1 (Ref)
	Illiterate	1 (25.0)	3 (75.0)	0.30	0.32 (0.01–4.36)
Mother's literacy status	Literate	137 (49.1)	142 (50.9)		1 (Ref)
	Illiterate	38 (52.8)	34 (47.2)	0.57	1.16 (0.67–2.01)
Family type	Nuclear	143 (49.1)	148 (50.9)		1 (Ref)
Joint	31 (51.7)	29 (48.3)	0.72	1.11 (0.61–2.00)
Monthly out-of-pocket expenditure (NRP)	≤600^d^	102 (45.7)	121 (54.3)		1 (Ref)
>600	73 (56.6)	56 (43.4)	0.05	1.54 (0.99–2.39)

a Computed using Chi-square test or Fisher exact test;

b significant OR at 95% CI;

c exact CI was computed due to the small number of respondents;

d classified using median monthly expenditure.

### Factors associated with smoking susceptibility

The percentage of smoking susceptibility among 352 respondents in the JD-HDSS was 49.7% (95% CI: 44.5–54.9). Table 2 summarizes the sociodemographic characteristics of the study population and shows smoking susceptibility in relation to each characteristic. Univariate analysis showed that being a male made the respondent more susceptible to smoking (OR=1.58, 95% CI: 1.04–2.42).

Table 3 describes the family and childhood environmental factors associated with smoking susceptibility. In univariate analysis, the odds of being susceptible to smoking is more than three times higher in adolescents who had seen a teacher smoke (OR=3.16; 95% CI: 1.77–5.65). Those adolescents who were involved in concerts/picnics (OR=3.07; 95% CI: 1.81–5.20) were more than three times likely to be susceptible to smoking. Similarly, adolescents who were exposed to pro-tobacco advertisements (OR=2.22; 1.41–3.49) were more likely to be susceptible to smoking. Being exposed to secondhand smoking, made adolescents twice as likely to be susceptible to smoking (OR=2.03; 95% CI: 1.12–3.67). Those adolescents whose friends’ smoked were more than two times likely to be susceptible to smoking (OR=2.02; 95% CI: 1.31–3.43). Furthermore, adolescents who had seen actors smoking on screen were more likely to be susceptible to smoking (OR=1.76; 95% CI: 1.11–2.79). Next, the odds of smoking susceptibility increased for adolescents whose other family members/relatives smoked (OR=1.61; 95% CI: 1.04–2.47). Surprisingly, the odds of smoking susceptibility increased with frequency of observing anti-smoking messages (OR=1.74; 95% CI: 1.13–2.68) as well as with exposure to an anti-smoking curriculum at school (OR=2.08; 95% CI: 1.05–4.12). The odds of smoking susceptibility decreased among adolescents whose family members discussed the harmful effects of smoking (OR=0.37; 95% CI: 0.23–0.59).

**Table 3:** Family and childhood environmental factors and potential association with smoking susceptibility among adolescents

		Susceptibility to smoking (*n*=175)	Non-susceptibility to smoking (*n*=177)		Unadjusted odds ratio (OR)
				
Variables	Responses	Number (%)	Number (%)	*P*^a^	(95% CI)
Parents smoke	No	66 (46.8)	75 (53.2)		1 (Ref)
	Yes	109 (51.7)	102 (48.3)	0.37	1.21 (0.79–1.86)
Sibling smokes	No	137 (48.9)	143 (51.1)		1 (Ref)
	Yes	23 (57.5)	17 (42.5)	0.31	1.41 (0.72–2.75)
Other family members/relatives smoke	No	63 (42.3)	86 (57.7)		1 (Ref)
Yes	109 (55.3)	88 (44.7)	0.03	**1.61 (1.04–2.47)^b^**
Family member ever asked you to light cigarettes	No	162 (48.4)	173 (51.6)		1 (Ref)
Yes	10 (71.4)	4 (28.6)	0.1	2.66 (0.8–8.68)
Family member ever asked you to purchase cigarettes	No	67 (46.9)	76 (53.1)		1 (Ref)
Yes	107 (51.4)	101 (48.6)	0.4	1.20 (0.78–1.84)
Friends smoke	No	100 (48.8)	105 (51.2)		1 (Ref)
	Yes	75 (67.0)	37 (33.0)	0.002	**2.02 (1.31–3.43)^b^**
Teachers smoke	No	117 (47.8)	128 (52.2)		1 (Ref)
	Yes	55 (74.3)	19 (25.7)	<0.001	**3.16 (1.77–5.65)^b^**
Exposure to secondhand smoke	Not exposed	21 (36.2)	37 (63.8)		1 (Ref)
	Exposed	154 (52.4)	140 (47.6)	0.018	**2.03 (1.12–3.67)^b^**
Involvement in extracurricular activities (quiz, debates, etc.)	No	132 (48.0)	143 (52.0)		1 (Ref)
Yes	43 (55.9)	32 (44.1)	0.09	1.46 (0.84–2.52)
Attendance at concerts/picnics with friends	Never	25 (29.4)	60 (70.6)		1 (Ref)
Sometimes/most of the times	150 (56.2)	117 (43.8)	<0.001	**3.07 (1.81–5.20)^b^**
Exposure to pro-tobacco advertisements	Few	45 (36.9)	77 (63.11)		1 (Ref)
A lot	130 (56.5)	100 (43.5)	0.001	**2.22 (1.41–3.49)^b^**
Seen actors smoking in movies or on TV	Sometimes	44 (40.0)	66 (60.0)		1 (Ref)
A lot	127 (54.0)	108 (45.9)	0.02	**1.76 (1.11–2.79)^b^**
Frequency of anti-smoking messages observed	A lot	89 (43.8)	114 (56.2)		1 (Ref)
Few/None	83 (57.6)	61 (42.4)	0.01	**1.74 (1.13–2.68)^b^**
Are there any anti-tobacco related topics in the school curriculum?	No	14 (34.1)	27 (65.9)		1 (Ref)
Yes	161 (51.9)	149 (48.1)	0.03	**2.08 (1.05–4.12)^b^**
Has anyone in your family discussed the harmful effects of smoking?	No	77 (66.4)	39 (33.6)		1 (Ref)
Yes	96 (42.1)	132 (57.9)	<0.001	**0.37 (0.23–0.59)**

Percentages are computed based on the row total and the total is not always 352 because of missing values or ‘do not know’ answer.

a Computed using Chi-square test or Fisher exact test;

b significant OR at 95% CI.

Table 4 shows the stepwise multiple logistic regression analysis. Among 10 significant factors (sociodemographic as well as family and childhood environmental factors) in univariate analysis, only four factors demonstrated significant association with smoking susceptibility (*P*<0.05). The model was statistical significant, that is, there was a significant relationship between smoking susceptibility and family and childhood environmental factors (LR chi 2^4^=38.36, Prob>Chi-square (4)<0.001) and therefore all other family and childhood risk factors including sex were dropped from the model (for *P*>0.05 and *P*<0.1). The highest adjusted *R* ^2^ was 0.18 indicating that collinearity was not present among the risk factors included in the model. Being exposed to pro-tobacco advertisements (AOR=2.49; 95% CI: 1.46–4.24) increased the odds of smoking susceptibility. Likewise, the odds of susceptibility increased among adolescents who had seen/noticed a teacher smoking (AOR=2.45; 95% CI: 1.28–4.68). The adolescents who participated in concerts/picnics were more likely to be susceptible to smoking than their counterparts who never participated in such activities (AOR=2.14; 95% CI: 1.13–4.04). Likewise, adolescents who were exposed to other family members smoking were more likely to exhibit increased susceptibility to smoking (AOR=1.76; 95% CI: 1.05–2.95).

**Table 4:** Stepwise multiple logistic regression for smoking susceptibility among adolescents

Variable	Susceptibility to smoking (*n*=175) number (%)^a^	Non-susceptibility to smoking (*n*=177) number (%)^a^	*P*^b^	Adjusted odds ratio (95% CI)
Exposure to pro-tobacco advertisements (a lot)	130 (56.5)	100 (43.5)	0.001	**2.49 (1.46–4.24)^c^**
Teachers smoke (yes)	55 (74.3)	19 (25.7)	0.006	**2.45 (1.28–4.68)^c^**
Participation in concerts/picnics with friends (yes)	150 (56.2)	117 (43.8)	0.02	**2.14 (1.13–4.04)^c^**
Other family members/relatives smoke cigarettes (yes)	109 (55.3)	88 (44.7)	0.03	**1.76 (1.05–2.95)^c^**

a Detailed information was given in Table 3;

b *p* values were computed from Chi-square test;

c significant AOR at 95% CI.

Table 5 shows the cumulative risk scores obtained from multiple logistic regression analysis. Compared to participants with ≤1 risk factor, adolescents exposed to two or more risk factors were more likely to be susceptible to smoking.

**Table 5:** Cumulative risk associated with smoking susceptibility

	Exposed	Susceptibility to smoking number (%)	Not susceptibility to smoking number (%)	*P*^a^	Odds ratio (OR) (95% CI)
Number of risk factors	≤1	20 (28.6)	50 (71.4)		1 (Ref)
2	55 (50.0)	55 (50.0)		**2.50 (1.26–4.99)^b^**
	3	70 (66.7)	35 (33.3)	*P*<0.001	**5.00 (2.47–10.22)^b^**
	4	24 (82.7)	5 (17.3)		**12.00 (3.69–44.70)^b^**

Percentages were computed based on row totals.

a *P* values were computed from Chi-square test;

b significant OR at 95% CI. Exact confidence interval was computed due to small number of respondents.

## Discussion

We explored the role of sociodemographic and environmental factors in smoking susceptibility in a low-income setting. Pierce et al. illustrates that smoking susceptibility is linked with age, sex, academic performance and exposure to other smokers (family members/friends), and family income (6). Likewise, other studies reveal that being a male, exposure to teacher and peer smoking, anti-smoking curricula, tobacco advertisements, and so on associate with smoking susceptibility (15, 17), which is consistent with our findings. Thus, adolescents’ from both high-and middle and low-income countries are influenced by several factors in their early stage of a smoking career (12, 13, 15–17). Indeed, the validity of smoking susceptibility has been tested only in the US and currently research is lacking on validity of smoking susceptibility in low- and middle-income countries (15). However, this is now possible based on the findings presented in this study.

We modified three different questionnaires related to smoking susceptibility with different time periods and with different response options so that respondents would feel comfortable to answer them. For example, the first question was ‘will you try to smoke soon’ (6). The word ‘soon’ caused confusion to the respondents and as a result, the period of time needed to be explained. That is why we included 6 months as a fixed period. Usually, a 6-month time period is useful to identify the percentage of adolescents who have ‘intention to smoke’ (36). Next, we included 5 years instead of 1 year in the third question of smoking susceptibility. Adolescents’ responses might not vary in a 6-month period or up to 1 year. After 5 years, those who were 14 years during the study period will be 19 and those who are 16 will be 21 years. These young adults who are aged 19–21 will still be vulnerable to initiate smoking. Thus, 5 years was included in our study. Guindon et al. measured smoking susceptibility by including 5 years as a time period (15). A study conducted in China used only one question, that is, ‘Do you foresee yourself taking up smoking in the next 12 months?’ (16). Those who responded positively were coded as susceptible to smoking. Thus, there is variation in smoking susceptibility questions and validity of tools is questionable. Lastly, based on our study, we recommend adding the response option ‘I do not know’ in future smoking susceptibility questionnaires.

Tobacco advertising is the strongest risk factor for smoking susceptibility in a peri-urban community in Nepal. A study from India revealed that tobacco advertisements significantly influenced the use of tobacco products among adolescents (47), despite bans against tobacco advertising (17). Similarly, Nepal has banned tobacco advertisements since 1998 but ineffective implementation of policies results in continuous exposure of adolescents (3, 4).

Unexpectedly, our univariate analysis revealed that anti-smoking messages and school curriculum associated positively with smoking susceptibility. The content and mode of delivery of anti-smoking messages could impact the outcome of messages (48). The school curriculum of a majority of our respondents (88%) included anti-smoking topics that associated positively with smoking susceptibility, possibly because knowledge-based interventions alone do not impact the health behavior of children and adolescents who lack social resistance skills (49, 50). Next, there might be the influence of socio-cultural factors. Recent data confirm that >50% of Nepalese males consume any form of tobacco (51). The consumption of tobacco associates with socio-cultural factors such as older age, illiteracy, marital status, occupation and residential region (51). In some Nepalese communities, alcohol use is culturally accepted during family gatherings and those who drink also smoke (52). When adolescents are exposed to smoking in such social environments, they may begin thinking about smoking differently and make plans to try smoking in the future, as they believe that smoking is a natural part of daily life. Therefore, it is crucial to understand the socio-cultural context of Nepal while designing tobacco control programs, which may influence adolescent's smoking behavior that may ultimately also influence their perception and reaction to anti-smoking initiatives.

Further, anti-smoking activities should incorporate health behavioral theories to enable a more effective tobacco control approach. In Finland, anti-smoking programs based on Bandura's social learning theory have been useful for understanding and preventing smoking initiation in adolescents (53). Bandura's theory explains that adolescents commonly initiate smoking due to social pressure at social gatherings. Therefore, teaching adolescents how to resist social pressure in such situations might aid prevention (53). A theory-based intervention may provide an effective approach to discouraging smoking initiation among adolescents. Likewise, the MYTRI project is another example of a school-based program that adopts a multi-strategy approach to reduce tobacco use among Indian adolescents (54).

Our results show that the likelihood of smoking susceptibility increased 2.45-fold when adolescents were exposed to teachers who smoke. A study in the Mahottray district of Nepal demonstrates that nearly 60% of school teachers use tobacco products inside school premises and 33% of them smoke cigarettes (55). Most of these teachers initiated tobacco use during their childhood and explained that imitation and peer pressure influenced them to start. Such behavior of a role model (in this case the teacher) influences students to initiate tobacco use. Moreover, the Health Education and Tobacco Intervention Project (HETIP) shows that many teachers and administrative staff who advocate non-smoking attitudes inside the schools nonetheless smoke tobacco when they are outside school premises (56). Indeed, HETIP reports correlation between the prevalence of student smoking and teacher smoking. Furthermore, awareness of the teacher of tobacco control policies might help prevent smoking initiation of adolescents.

Our findings suggest that smoking by family members strongly predicts smoking susceptibility among adolescents. Other studies from Nepal show that family members smoking reinforce factors for tobacco use among adolescents (25, 57). Further, the magnitude of exposure to on-screen smoking by actors significantly predicts smoking initiation among adolescents (58, 59). However, such exposure was not statistically significant in our final model. Therefore, family members and actors should act as role models for children and promote anti-tobacco use. Restrictions of tobacco use at home and other public places might also discourage adolescents not to smoke through ‘smoking is socially unacceptable’ messages.

Our results reveal a positive association between smoking susceptibility and adolescents’ attendance at concerts/picnics. During the gathering, people rarely discuss the harmful effects of smoking and drinking. The participation in concerts/picnics means not only gatherings with friends and family members but also to have a drink and smoke. In such environments, adolescents have opportunities to initiate smoking because they like to imitate seniors and peers who smoke. As described in Bandura's theory of social learning, adolescents learn the meaning of smoking in the social context of interaction (11, 38).

Parallel with different health behavior theories (8–11), our study demonstrates that exposure to family and childhood environmental factors (i.e. reasons) such as pro-tobacco advertisements and smoking by family members or teachers encourages adolescents to initiate smoking (i.e. behavior) when they have easy access to cigarettes and opportunities to smoke in the absence of parents (e.g. during concerts/picnics). Susceptibility is less likely in adolescents when family members discuss the harmful effects of smoking.

Most tobacco-related studies that measure the role of sociodemographic variables report varying results (60). Our multiple regression analysis detected no association between sociodemographic variables (e.g. age, sex, ethnicity, and parental education) and smoking susceptibility. We found that sex is associated with smoking susceptibility in univariate analysis but diluted in multivariable analysis. Other sociodemographic variables did not show any differences between susceptible and non-susceptible adolescents. The rapid urbanization changes people ways of living through exposure to modifiable risk factors including smoking (61). It has a significant impact on demographic and social structures when a paradigm shift occurs from traditional to modern lifestyles with improved earning capacity (30, 61). A recent study in India revealed an association between modern lifestyle and tobacco use among adolescents (62). Additionally, there is a changing pattern of socio-economic status and tobacco use among adolescents over a time; the prevalence of tobacco use is higher among lower (vs. higher) socio-economic status students at baseline but after 2 years, both groups exhibit equal smoking prevalence (63). Urbanization, changing lifestyle pattern and changing socio-economic status are possible reasons for no difference in sociodemographic factors in susceptibility to smoking among adolescents in our study, which was conducted in a peri-urban area of Nepal.

Several studies report that having a friend who smokes is associated with smoking behavior of adolescents but we found no such correlation in our peri-urban setting in Nepal. Moreover, a Texan study suggests that peer pressure does not predict smoking susceptibility because susceptibility to smoking describes influence of peer pressure and intention to smoke in the future (14).

In our study, nearly 30% respondents were excluded due to non-responses as they mainly answered ‘I do not know’ or gave no response to some questions. Some of the study variables were significantly different between responders and non-responders. For example, non-responder females did not share their smoking behavior in comparison to males. Earlier tobacco research in Nepal has shown that smoking behavior is common among men (23–27). Females may be reluctant to reveal their smoking behavior in Nepalese society because of cultural restriction. Thus, females might underreport their smoking behavior because of social stigma. Furthermore, there was a significant difference in some of the sociodemographic and family and childhood environmental characteristics between responders and non-responders. Based on this information, it is difficult to explain the adolescents’ smoking behaviors. We are also not sure whether they have underreported or are not willing to respond to certain questions. Thus, the larger non-response rate can reduce sample size and thereby increase the standard error. For example, if we take 485 non-smoking adolescents including non-responders, standard error of smoking susceptibility was 0.022 while taking 352 adolescents, the standard error for smoking susceptibility changed to 0.026.

There are several limitations of our study. Though susceptibility to smoking is a valid measure of experimentation, its validity has not been tested in our study. However, Pierce et al. determined that susceptibility to smoking is a good predictor of experimentation (6).

There is also a possibility of recall bias when questions are asked about pro-tobacco advertisements and anti-smoking messages because both occurred simultaneously. Local enumerators collected information during interviews, which might lead to the respondents being reluctant to disclose their smoking behavior due to social image. Thus, there might be underreporting of smoking behavior which leads to social desirability bias. Perception of risks and the harmful effects of tobacco smoking could influence the initiation of smoking. However, we could not cover all influencing factors in this study. Furthermore, respondents were selected from a peri-urban area near Kathmandu and may not reflect all peri-urban areas in Nepal. As our study was cross-sectional, we could not establish temporal or causal associations.

Despite these limitations, our results provide important information about correlates of smoking susceptibility of Nepalese adolescents. This is a community-based study that examines factors associated with smoking susceptibility. Additionally, our sample size allowed precise estimates of effects and valid comparisons, and concurring with a US study (13), we demonstrate that exposure to multiple risk factors increased smoking susceptibility. Because we adopted a probability sampling technique, our sample is representative of the population of the study area. Further, the findings from our study have several implications for developing effective intervention programs for Nepalese adolescents living in peri-urban areas of Nepal. Smoking intervention programs for Nepalese adolescents should: (a) focus not only on smoking and non-smoking but also on those who are susceptible to smoking; (b) provide awareness on several family and environmental factors associated with smoking; (c) teach effective smoking refusal skills in relation to peer pressure; (d) provide training to resist pro-tobacco advertisements; (e) involve non-smoking role models; (f) family members should discuss the harmful effects of smoking with their children. Besides effective intervention programs, future research should be conducted with a large sample size in both urban and rural settings that include other influencing factors like perceptions of risks and harm.

## Conclusions

Smoking susceptible adolescents are prevalent in the JD-HDSS, a peri-urban community of Nepal. Factors that increased susceptibility to smoking among Nepalese adolescents included exposure to pro-tobacco advertisements, smoking by teachers, attending concerts/picnics with friends, and other family members (not father/mother) and relatives who smoke cigarettes. Therefore, to be effective, future intervention efforts should be focused on family and childhood environmental factors with emphasis on: impact of role models smoking, refusal skills in social gatherings, and discussing the harmful effects of smoking with family members and during gatherings with friends.

## Appendix 1

**Table:** Sociodemographic characteristics of responders and non-responders

		Responder (n=352)	Non-responder (n=133)	Total (n=485)	
			
Variable	Responses	Number (%)	Number (%)	Number (%)	*P*#
Sex	Female	161 (67.9)	75 (31.8)	236 (100)	
	Male	191 (76.7)	58 (23.3)	249 (100)	**0.04**
Age	16	107 (30.4)	32 (23.0)	139 (100)	
	15	120 (69.0)	54 (31.0)	174 (100)	
	14	125 (72.7)	47 (27.3)	172 (100)	0.29
Ethinic group	Upper caste	196 (75.1)	65 (24.9)	261 (100)	
	Relatively advantaged	133 (70.4)	56 (29.6)	189 (100)	
	Indigenous and socially disadvantaged	23 (65.7)	12 (34.2)	35 (100)	0.46
Education Status (Grade)	Higher secondary (11–12)	90 (70.3)	38 (29.6)	128 (100)	
Secondary (9–10)	229 (76.6)	70 (23.4)	299 (100)	
	Lower Secondary (6–8)	30 (51.7)	28 (48.7)	58 (100)	0.69
Wealth quintiles	Lowest	67 (95.7)	3 (4.3)	70 (100)	
	Second	65 (63.1)	38 (36.9)	103 (100)	
	Middle	65 (54.5)	55 (45.8)	120 (100)	
	Fourth	75 (70.7)	31 (29.4)	106 (100)	
	Upper	69 (80.2)	17 (19.7)	86 (100)	0.24
Father's occupation	Service	143 (71.9)	56 (28.1)	199 (100)	
	Business	82 (71.9)	32 (28.1)	114 (100)	
	Farmer	101 (72.7)	38 (27.3)	139 (100)	
	Retired/unemployed	19 (86.4)	3 (13.6)	22 (100)	
Mother's occupation	Service	17 (60.7)	11 (39.3)	28 (100)	0.53
	Agriculture	77 (77.8)	22 (22.2)	99 (100)	
	Housework	224 (73.0)	83 (27.0)	307 (100)	
	Business	33 (67.3)	16 (32.7)	49 (100)	0.26
Father's literacy status	Literate	340 (72.6)	128 (27.4)	468 (100)	
	Illiterate	4 (66.7)	2 (33.3)	6 (100)	
Mother's literacy status	Literate	340 (72.6)	128 (27.4)	468 (100)	0.74
	Illiterate	4 (66.7)	2 (33.3)	6 (100)	
Family type	Nuclear	291 (74.6)	99 (25.4)	390 (100)	
	Joint	60 (63.8)	34 (36.2)	94 (100)	**0.03**
Monthly out-of-pocket expenditure (NRP)	≤600	223 (68.6)	102 (31.4)	325 (100)	
>600	129 (80.6)	31 (19.4)	160 (100)	**0.005**

Percentages are computed based on the row total and total is not always 485 because of missing values or “do not know” answer. **#** computed using chi-square test or fisher exact test.

## Appendix 2

**Table:** Family and childhood environmental factors among responders and no responders

		Responder (n=352)	Non-responder (n=133)	Total (n=485)	
			
Variables	Responses	Number (%)	Number (%)	Number (%)	*P*#
Parents smoke	No	141 (40.1)	64 (48.1)	250 (100)	
	Yes	211 (59.9)	69 (51.9)	280 (100)	0.60
Sibling smokes	No	40 (74.1)	14 (25.9)	54 (100)	
	Yes	280 (70.2)	119 (29.8)	399 (100)	
Other family members/relatives smoke	No	149 (68.3)	69 (31.9)	218 (100)	
	Yes	197 (76.1)	62 (23.9)	259 (100)	0.06
Family member ever asked you to light cigarettes	No	335 (73.1)	123 (26.9)	458 (100)	
Yes	14 (60.9)	9 (39.1)	23 (100)	0.20
Family member ever asked you to purchase cigarettes	No	143 (68.4)	66 (31.6)	208 (100)	
Yes	208 (75.6)	66 (24.4)	276 (100)	0.07
Friends smoke	No	205 (71.9)	80 (28.1)	285 (100)	
	Yes	112 (82.4)	24 (17.6)	136 (100)	**<0.001**
Teachers smoke	No	245 (73.6)	88 (24.6)	333 (100)	
	Yes	74 (84.4)	18 (19.6)	92 (100)	0.20
Exposure to secondhand smoke	Not Exposed	294 (73.5)	106 (26.5)	400 (100)	
Exposed	58 (68.2)	27 (31.8)	85 (100)	0.32
Involvement in extracurricular activities (quiz, debates, etc.)	No	275 (72.8)	103 (27.2)	378 (100)	
Yes	75 (72.8)	28 (27.2)	103 (100)	0.99
Attendance at concerts/picnics with friends	Never	85 (64.4)	47 (35.6)	132 (100)	
Sometimes/Most of the times	267 (75.6)	86 (24.4)	353 (100)	**0.01**
Exposure to pro-tobacco advertisements	Few	122 (76.7)	37 (23.3)	159 (100)	
A lot	230 (70.6)	96 (29.4)	326 (100)	0.15
Seen actors smoking in movies or TV	Sometimes	110 (82.1)	24 (17.9)	134 (100)	
A lot	235 (69.7)	102 (30.3)	337 (100)	**0.006**
Frequency of anti-smoking messages observed	Few/None	144 (64.0)	81 (36.0)	225 (100)	
A lot	203 (80.2)	50 (19.8)	253 (100)	**<0.001**
Are there any anti-tobacco related topics in the school curriculum?	No	41 (63.1)	24 (36.9)	65 (100)	
Yes	310 (74.2)	108 (25.8)	418 (100)	0.06
Has anyone in your family discussed the harmful effects of smoking?	No	116 (77.9)	33 (22.1)	149 (100)	
Yes	228 (69.7)	99 (30.3)	327 (100)	0.09

Percentages are computed based on the row total and total is not always 485 because of missing values or “do not know” answers. **#** computed using chi-square test or fisher exact test.
